# The marine geological imprint of Antarctic ice shelves

**DOI:** 10.1038/s41467-019-13496-5

**Published:** 2019-12-10

**Authors:** James A. Smith, Alastair G. C. Graham, Alix L. Post, Claus-Dieter Hillenbrand, Philip J. Bart, Ross D. Powell

**Affiliations:** 1 British Antarctic Survey, High Cross, Madingley Road, Cambridge, CB3 0ET UK; 2 College of Life and Environmental Sciences, University of Exeter, Exeter, UK; 3 Geoscience Australia, GPO Box 378, Canberra, ACT 2601 Australia; 4 Louisiana State University, Department of Geology and Geophysics, Baton Rouge, 70803 USA; 5 Department of Geology and Environmental Geosciences, Northern Illinois University, DeKalb, IL USA; 6 Present Address: College of Marine Science, University of South Florida, St Petersburg, FL 33701 USA

**Keywords:** Climate sciences, Environmental sciences, Ocean sciences, Scientific community

## Abstract

Reductions in the thickness and extent of Antarctic ice shelves are triggering increased discharge of marine-terminating glaciers. While the impacts of recent changes are well documented, their role in modulating past ice-sheet dynamics remains poorly constrained. This reflects two persistent issues; first, the effective discrimination of sediments and landforms solely attributable to sub-ice-shelf deposition, and second, challenges in dating these records. Recent progress in deciphering the geological imprint of Antarctic ice shelves is summarised, including advances in dating methods and proxies to reconstruct drivers of change. Finally, we identify several challenges to overcome to fully exploit the paleo record.

## Abstract (web summary)

The recent collapses of ice shelves in Antarctica due to warming make it essential to understand past ice shelf conditions and mechanisms. Here Smith and colleagues review the latest progress in deciphering the geological imprint of Antarctic ice shelves via sediments, landforms and proxy indicators.

## Introduction

**Fig. 1:**
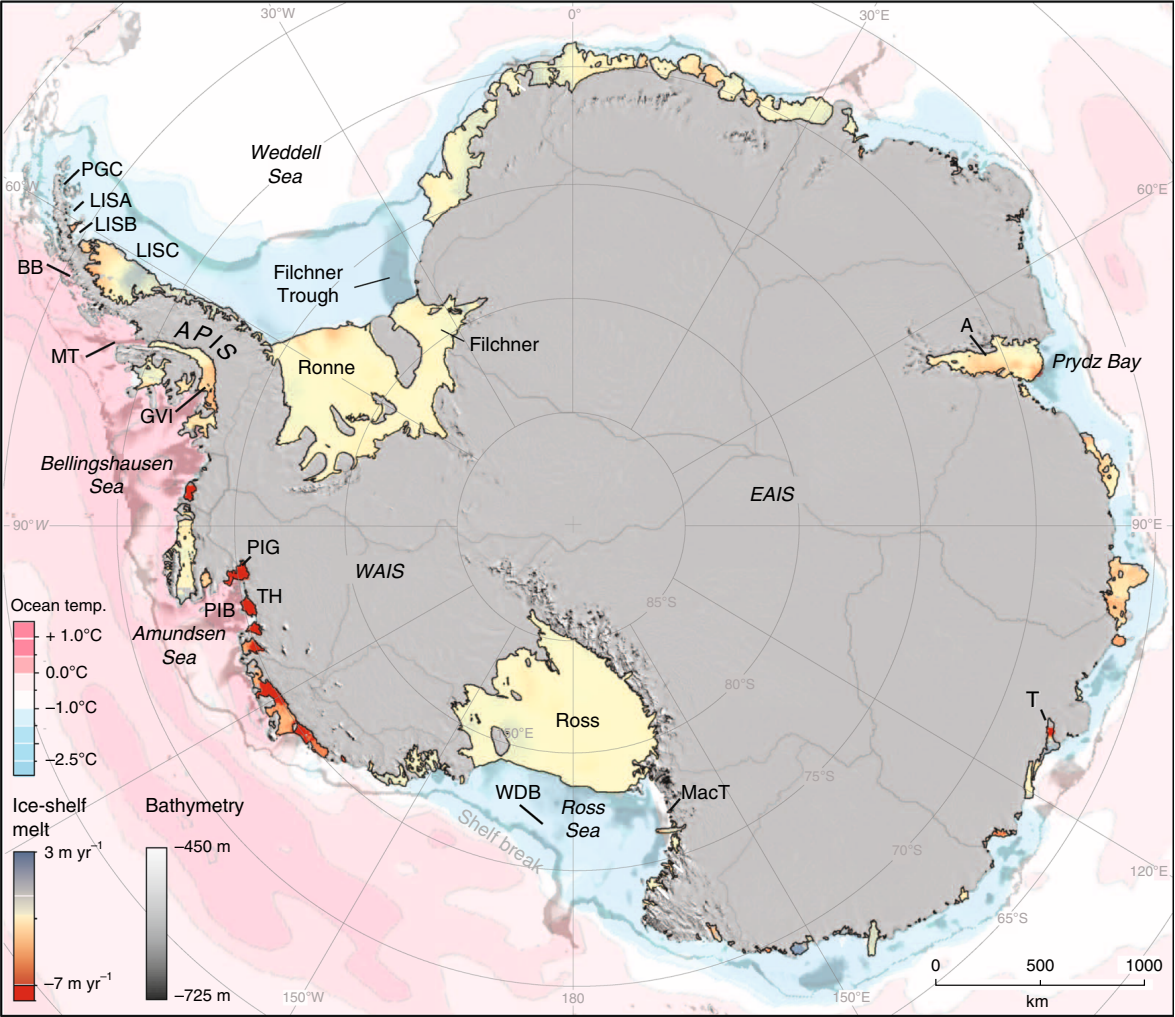
Rate of change of Antarctic ice-shelf and ice-sheet thickness^1^. Estimated average seafloor potential temperatures (in °C) from the World Ocean Circulation Experiment Southern Ocean Atlas^145^ (pink to blue) are overlaid on continental shelf bathymetry (in metres)^146^ (greyscale, landward of the continental-shelf break). APIS Antarctic Peninsula Ice Sheet, WAIS and EAIS West and East Antarctic Ice Sheets, PGC Prince Gustav Channel, LISA Larsen A, LISB Larsen B, LISC Larsen C, BB Barilari Bay, MT Marguerite Trough, GVI George VI Ice Shelf, PIG/PIB Pine Island Glacier/Bay, TH Thwaites, MacT Mackay Glacier Tongue, WDB Whales Deep Basin,T Totten, A Amery.

The majority of grounded ice in Antarctica drains through the ice sheet’s peripheral ice shelves making them a critical component of its mass balance^1,2^. The rapid collapse of the Larsen B Ice Shelf in 2002 not only demonstrated the sensitivity of the Antarctic cryosphere to recent warming^3^, but also confirmed earlier theoretical work that ice shelves act to buttress inland ice^4^. The near-instantaneous acceleration of outlet glaciers following collapse resulted in greater land-ice discharge and contribution to sea-level rise^5,6^. Breakup of ice shelves along the Antarctic Peninsula has been linked to atmospheric warming since the late 1800s together with specific mechanisms, e.g., hydrofracture in water-filled crevasses^7–9^. Some Peninsula ice shelves, together with those along the West Antarctic Ice Sheet (WAIS) Pacific margin (Fig. 1), have also thinned in response to enhanced submarine melting from intrusion of warm circumpolar deep water (CDW)^1,10^ onto the continental shelf which weakens buttressing, resulting in accelerated ice flow^1^. In the Amundsen Sea sector of WAIS, where mass loss due to ice shelf thinning has increased substantially over the past four decades^11^, numerical models suggest that runaway deglaciation of the ice sheet is possible and might already be underway^12^. Ocean-driven thinning is also detected for key ice shelves fringing the East Antarctic Ice Sheet (EAIS)^13^, suggesting that this region is also susceptible to rapid and large-scale ice loss^14^, and together with contributions from the Antarctic Peninsula and WAIS could drive significant future sea-level rise^11,15^.

In addition to an improved understanding of the sensitivity of Antarctic ice shelves to ocean and atmospheric forcing, there is a growing awareness that some currently observed changes were instigated in the recent past^16^ and in some cases, ice shelves might have been pre-conditioned to collapse through many thousands of years of thinning^17^. This has highlighted the need for detailed reconstruction of ice-shelf history at a range of time scales so that the relative roles of contemporaneous ocean–atmosphere forced-change and/or a continuing response to an earlier perturbation in driving ice shelf loss can be fully assessed. Furthermore, the geological record can serve as an important analogue for recent and future changes and particularly dynamic thinning, where the impacts of ice-shelf removal on grounded ice further upstream can be fully explored and used to constrain model simulations. Such records will not only help decipher the drivers of change but are also important for initialising models which aim to predict future change^15^. The more important the long-term behaviour, the more likely the model will be required to run over longer time scales^18^.

In this review, we examine sediments, landforms and available proxy indicators associated with ice-shelf presence, absence and collapse, by utilising new information obtained from direct access^10,16,19–22^ and the considerable theoretical and observational advances that have been made by studying recently exposed and former sub-ice-shelf environments^17,23^. We consider how this information can constrain key boundary conditions relevant for modelling studies, such as ice shelf extent, thickness and cavity geometry, and review the methods currently available to assess the rates and drivers of past retreat. Finally, key gaps in our understanding of these systems are identified as areas for future research. Past or ‘paleo’ largely refers to changes since the Last Glacial Maximum (LGM; 19–23 kyr) because, unlike previous glaciations, the sediments and landforms are comparatively well-preserved and consequently easier to interpret and date accurately. The concepts and applications are also relevant to more ancient glaciations, which is particularly important as future climate conditions might differ from the LGM/Holocene.

## Observations beneath contemporary ice shelves

**Fig. 2:**
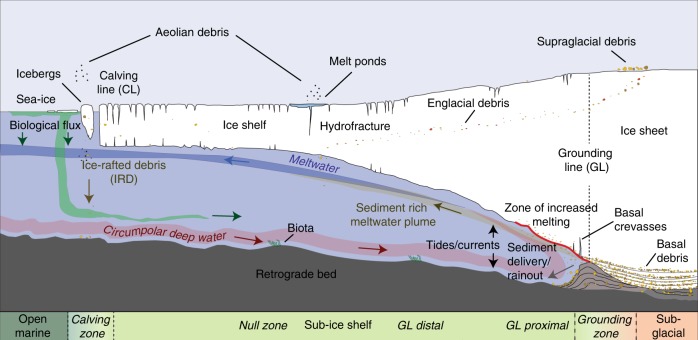
Simplified view of an Antarctic ice shelf influenced by warm water inflow (not to scale). Inflowing warm water drives enhanced melting at the ice-shelf base in the vicinity of the grounding line (GL) and results in outflowing meltwater that is cooler and fresher.

The processes operating beneath modern ice shelves (Fig. 2) are critical to understanding the signatures left by past ice shelves yet they remain poorly characterised due to the logistical challenges associated with direct access to sub-ice shelf cavities. Observations made by a remotely operated vehicle (ROV) in the 1990s^24^ beneath Mackay Glacier Tongue, fed by an outlet glacier of the EAIS flowing into the Ross Sea (Fig. 1), remain the most comprehensive. At the grounding line (GL; Fig. 2), glacigenic debris up to 20-m-thick was observed in vertical faces of basal crevasses, consisting of discontinuous layers of diamicton or clast-rich mud interlayered with cleaner ice. Morainal banks were observed against the vertical face of such crevasses, formed as sediment was squeezed and pushed as the GL oscillated due to tidal flexure. Englacial debris was observed to be spatially patchy, with only a few layers cms to 1-m-thick present in calved icebergs and at the sides of the glacier tongue. Although basal melting was detected, no glacifluvial deposits or conduits indicative of extensive releases of meltwater were observed, suggesting a leaky drainage system through the till or between the till-glacier sole interface (cf. refs. ^25,26^). This contrasts with Arctic systems where meltwater-related processes are much more common^27^. Concentrations of suspended particulate matter increased near the GL, originating from either meltwater at the ice-bed interface or by re-suspension of seafloor sediments caused by increasing current velocities within a confined cavity. Up to 300 m away from the GL, a soft glacimarine drape consisting of clast-rich sandy-mud (shelfstone mud^28^) and diamicton with a poorly sorted texture similar to that of its basal debris source was imaged. The poor sorting indicated little subsequent sorting during rain-out from the ice shelf base. A key observation was that the drape is also locally patchy reflecting variations in rainout process and modification by ocean currents and/or by meltwater discharge.

More recent autonomous underwater vehicle (AUV) missions have prioritised the measurement and characterisation of ocean-water properties^10,29^. An exception to this are the AUV missions beneath Pine Island Glacier (PIG) ice shelf using Autosub, which not only revealed detailed information about the properties of the ocean and cavity geometry^10^ but also the morphology of the seafloor and ice base^21,30^. Inflowing CDW is warm and salty and contrasts with the melt-laden outflow that has been cooled and diluted by the addition of fresh water (Fig. 2). Under PIG, this outflow carries a signature of increased light attenuation arising from sediment-laden meltwater discharge or the melting of debris-rich basal ice immediately downstream of the GL. Sub-ice shelf sediment cores recovered at three locations along a prominent seafloor ridge beneath PIG—which is thought to have been the last stable pinning point as the PIG GL retreated landward—revealed the upper 4–6 cm consisted of finely laminated muds, consistent with a sediment-plume source^16^.

**Fig. 3:**
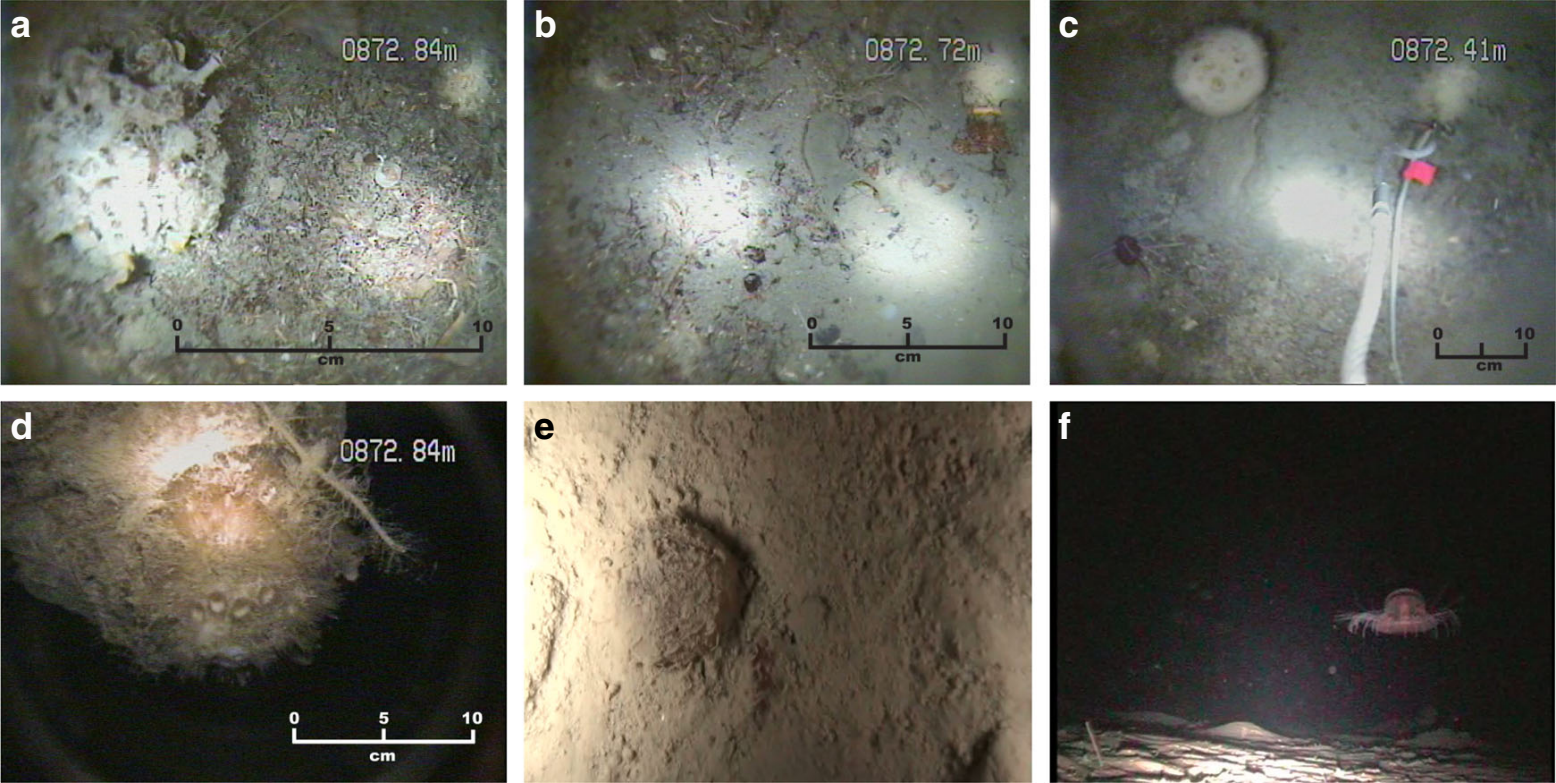
Seafloor biota imaged beneath the Amery Ice Shelf illustrating variations in ocean current circulation beneath the ice shelf. **a**–**d** Diverse community dominated by sessile filter feeders at sites of inflowing bottom currents, 100 km from the calving front. **e** Deposit-feeders 200 km from the calving front associated with inflowing bottom currents. **f** Mobile scavengers imaged 160 km from the calving front in an area of weak bottom current circulation. For details of biota, see ref. ^42^. Images © Martin Riddle/Australian Antarctic Division.

Access to ice shelf cavities via hot-water drilled boreholes are more numerous and allow direct measurements of critical boundary processes operating at the ice shelf base^31^ and seafloor. Measurements include sediment sampling^16,19,20^ and analyses of the biological communities living under the ice^32–34^, in the water column^35^, and on and within the seabed^36,37^. Early work on sub-ice shelf environments considered these regions unlikely to support living biota due to the absence of primary producers in settings devoid of light. Photographs from beneath the Ross Ice Shelf provided the first evidence for living biota beneath an Antarctic ice shelf hundreds of kilometres landward of ocean water, but also confirmed the low productivity of this environment with a relatively depauperate benthic assemblage comprised solely of mobile scavengers^32,38^. Fossilised benthic organisms in debris bands on the surface of the McMurdo Ice Shelf were used to infer the existence of a living benthic community beneath this ice shelf^39,40^, with a relatively impoverished community more recently imaged from the seaward part of this ice shelf^41^. In contrast, video footage collected 100 km from the calving front of the Amery Ice Shelf revealed a more diverse epifauna assemblage, including 29 distinct benthic taxa, mostly sedentary filter feeders, within an area of only 1–2 m^2^ (ref. ^36^) (Fig. 3a–d). Lower diversity communities, dominated by mobile scavengers, occurred 160 and 200 km upstream from the calving front of the Amery Ice Shelf^42^ (Fig. 3e, f). This work fundamentally changed our perception of sub-ice-shelf environments and specifically of the marine geological signature of ice shelf presence. Here the diverse benthic community could be mistaken for other low-light environments, such as coastal regions with perennial sea-ice cover^43^ and deep-water shelf regions below the photic zone^44–46^ if the context was not already known^42^.

## The marine geological signature of ice shelf presence

A fundamental requirement for paleo-ice shelf studies is to reconstruct their geometry, including extent and thickness, together with temporal changes in these properties. In this section, we summarise the key sediment types, proxies and landforms used to determine ice shelf presence, absence and collapse (Fig. 2). Epishelf lakes as recorders of ice shelf history^47,48^ are not covered here (see instead ref. ^49^).

### Sediment facies and proxies

**Fig. 4:**
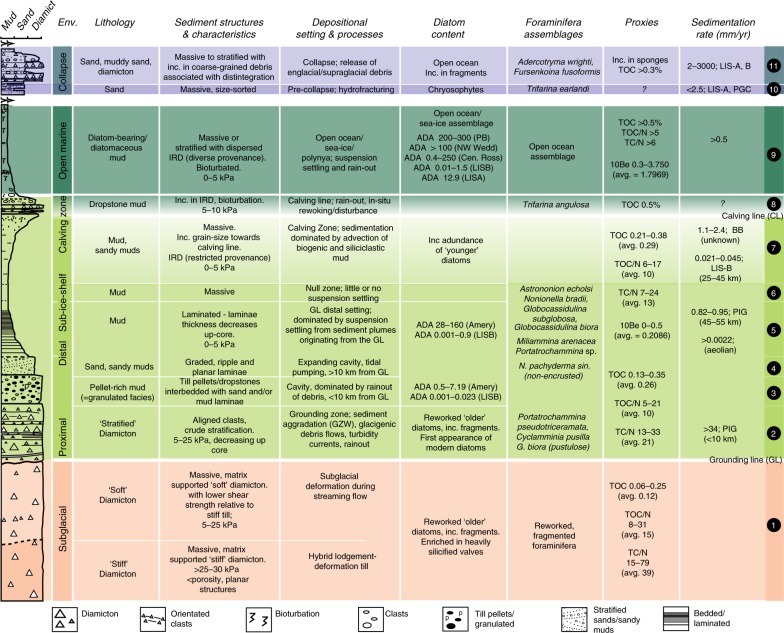
Idealised sedimentary sequence deposited under a retreating ice shelf (modified from refs. ^53,147^). This full succession is rarely preserved, although the general transition from coarse-grained grounding line (GL) proximal to finer-grained grounding line distal sediments is typical. A ‘collapse facies’ is summarised in the upper panel and can occur anywhere within the ‘sub-ice-shelf’ succession. Black numbered circles (right column) refer to the ‘sediment elements’ shown in Fig. 5. LISA, LISB Larsen A, B ice shelf, PGC Prince Gustav Channel Ice Shelf, BB Barilari Bay, PIG Pine Island Glacier, PB Prydz Bay. Sedimentation rates from refs. ^16,23,118^; number in parenthesis is distance from GL. Absolute diatom abundance (ADA; millions of valves/gram sediment)^17,22,63,64,96^. Percentage total organic carbon (TOC)/Nitrogen (N) and total carbon (TC)/N data^16,63^ together with unpublished data from George VI and Larsen C sub-ice-shelf cores. Berylium-10 (×10^9^ atoms/g) data^100–102^. Diatom assemblage data^61,73^, foraminiferal assemblage and morphotype data are compiled from refs. ^59,72,76–78,80,81^.

**Fig. 5:**
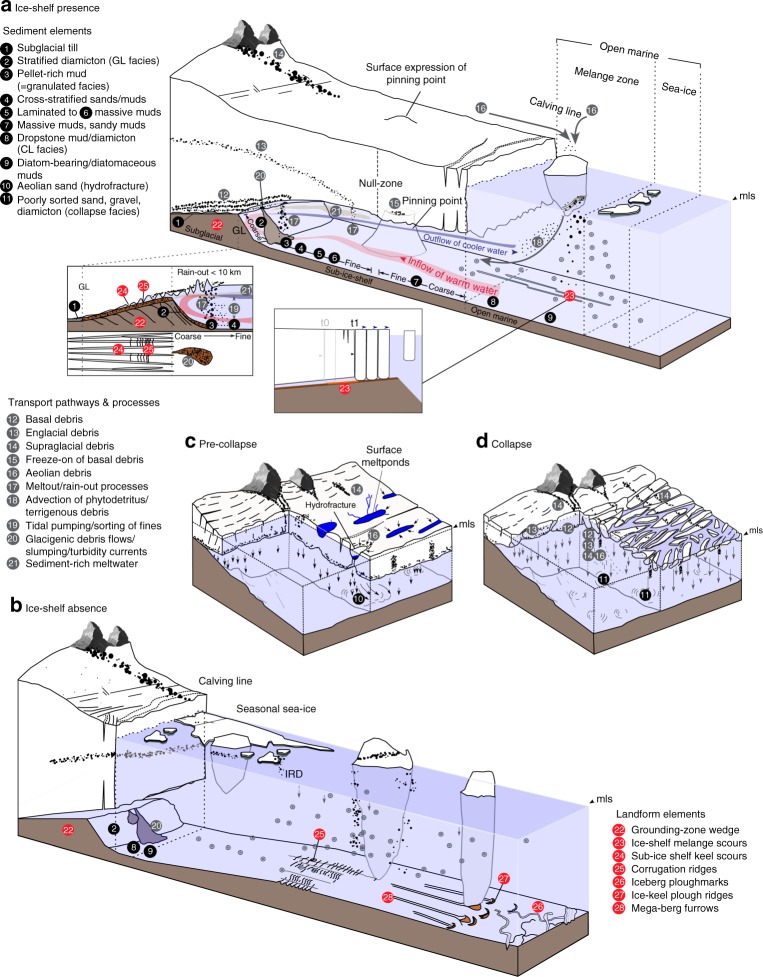
Conceptual model of the sediments and landforms associated with **a** ice shelf presence, **b** absence, **c** pre-collapse and **d** collapse (panel **c**, **d** modified from ref. ^94^). The characteristics of each sediment element is summarised in Fig. 4. Landform elements are summarised in Table 1. msl mean sea level.

Sub-ice-shelf sediments and proxies within them provide the most direct evidence of changing ice cover. With the exception of the area proximal to the GL (<10 km), sediments in sub-ice-shelf environments are characterised by low depositional rates (relative to other glacimarine environments) and low amounts of biogenic matter. Sedimentation is controlled by proximity to the sediment sources—the GL and calving line (Fig. 2)—but is also heavily influenced by marine currents, the spatial variability of debris entrainment in the ice as well as basal melting and freezing processes that can either enhance or retard debris release and rainout onto the sea floor^23^. Idealised models for sedimentation beneath Antarctic ice shelves^25,50–57^ tend to consider a retreating GL and calving line, and typically comprise a basal diamicton (subglacial facies representing ice-advance; Fig. 4) overlain by a sequence of sub-ice shelf sediments consisting of stratified diamicton and/or pellet-rich diamicton (‘granulated facies’), massive to stratified, well-sorted glacimarine sands and muds which first fine upwards into a ‘null zone’ before coarsening upwards again. The latter coarsening indicates the approaching calving line and is usually characterised by higher concentrations of both biogenic matter and silty to sandy detritus advected under the ice shelf from the open ocean (Figs. 4 and 5). This is capped by an iceberg-rafted diamicton deposited at the ice-shelf front, which in turn is overlain by a dropstone-bearing biogenic mud deposited in a seasonally open marine setting.

While subsequent work has demonstrated the general applicability of these stratigraphic models, especially as a robust framework to infer GL proximal to distal sedimentation, it has also highlighted the variable and heterogeneous nature of sub-ice-shelf sediments^25,58,59^. Ultimately, any of the aforementioned sedimentary units may be muted, amplified or entirely absent, and the formation/preservation of a particular unit depends on the time scales over which the various depositional processes operate as well as the size of the ice shelf. Sediments associated with short-lived phases of ice shelf cover will differ from those deposited beneath a large ice shelf system that exists for many thousands of years. It is also worth noting the potential differences between glacier tongues vs. ice shelves. Although many of the processes, facies and landforms are likely to be similar, their lateral extent and overall footprint will differ, as will the biogenic signature within the sediments. For example, because ocean currents are more likely to circulate freely under a glacier tongue, sediments deposited away from the GL will be similar to outer parts of a larger ice shelf system.

Despite this heterogeneity, a consistent feature in most sub-ice-shelf sediments is the dominance of a coarse-grained GL-proximal facies (diamicton, gravel-rich and sand-rich sediments) and a lack or paucity of subaqueous outwash^60^. The latter reflects limited subglacial meltwater discharge (relative to Arctic ice shelves^51^) potentially allowing effective discrimination between polar ice-shelf and more temperate or sub-polar tidewater glacimarine regimes. However, coarse-grained facies occur directly above subglacial tills across much of the Antarctic shelf and do not necessarily reflect ice shelf cover. In this context, several authors ascribe ‘granulated facies’, consisting of muddy-gravel, rich in granules or ‘till pellets’ (pelletized-mud^53^) (Fig. 4), solely to sub-ice-shelf deposition^54^. Deposition of the granulated facies has been explained by a combination of regelation freezing near to the GL and re-melting of this basal debris in a sub-ice shelf setting^53^. Sub-ice shelf ocean currents and/or tidal pumping (Fig. 5) sort the finer-grained detritus released by melting from the ice shelf base, which can result in coarse lag deposits with a ‘granulated texture’. Micropaleontological studies indicate that diatoms or diatom fragments within the granulated facies tend to be enriched in heavily silicified taxa, with fewer larger or pennate taxa^61^, but this applies more generally to GL-proximal sediments. The genesis of till pellets is thought to reflect mechanical shearing of till into pellets or alternating thermal conditions which promote rotation of fine-grained till aggregates^62^. While both formation mechanisms can plausibly result in the rainout of the pellets in a sub-ice-shelf cavity, such pellets are also common in the upper part of the deforming subglacial layer meaning their presence in glacial sequences is not always diagnostic of a sub-ice-shelf cover.

The transition from coarse- to fine-grained sediments typically reflects increasing distance from the GL, and on a retrograde seabed, an enlarging sub-ice cavity^16^. The presence of cross-bedded sands directly above the coarse-grained facies has been interpreted to reflect strong traction currents in a widening, but relatively narrow ocean cavity (Figs. 4 and 5)^20,24,54,63^. With increasing distance from the GL, the gravel and sand rich sediments are eventually replaced by sometimes thick sequences of laminated to massive muds and sandy muds deposited by sediment-laden meltwater plumes^16,25,26,64^, observed to extend 250 km from the GL^25^. The overall thickness of this unit is primarily a function of time, but also the competing influence of sediment delivery, and strength and longevity of ocean currents (which can be tidally generated)^16,23,64^. On large ice shelves, where the GL and calving line can be separated by >100 km, the laminated muds grade into massive muds with progressive distance from the GL before eventually reaching a theoretical zone where sedimentation is almost zero (‘null zone’ in Figs. 2, 4 and 5). The calving line, and thus the seaward limit of the ice shelf, can be identifiable by a distinct maximum in ice-rafted debris (IRD)^53,59,63^ and elevated diatom/organic contents^65^ (Fig. 4). Trapping of sea-ice and icebergs at the calving line increases their residence time at the ice-shelf front, leading to accumulation of IRD on the seafloor over time. Debris can include aeolian components (Fig. 5), while biogenic material is delivered by landward advection beneath the ice shelf through inflowing currents^53^. The calving line, however, is not constant through time due to periodic iceberg calving, and this might be recognised by sandier or dropstone-rich layers with higher contents of biogenic detritus. Thicker sequences of dropstone-rich mud is expected if the calving line is stable over a longer period of time (10^2^–10^3^ years).

The absence of IRD within the largely terrigenous laminated to massive muds has traditionally been used as an important indicator for ice shelf cover based on the assumption that larger ice-stream fed ice shelves are devoid of coarse particle sizes beyond the GL^50^. However, several studies have described poorly sorted debris at the surface of and within large ice shelves^66–68^ which either remains on the ice shelf surface and is later deposited as IRD at the calving line^67^ or is entrained into the ice shelf by inflowing glaciers^69^ before melting out at its base. Coarse-grained debris can also freeze to the base of an ice shelf as it grounds on pinning points during phases of advance, ice shelf thickening or locally lowering sea-level^56^. This debris is subsequently redistributed as the ice shelf unpins and is exposed to basal melting^24,56^ or, in some unique cases, can migrate to the surface as a result of high rates of surface ablation^40^. An expected corollary of this is that the sediment flux and grain size will vary over time, depending on which ice layer is being melted at the base^24^. However, debris incorporated into the ice shelf englacially or from freeze-on is likely to be locally sourced, making it distinguishable from calving-line diamictons, where IRD can be sourced from icebergs originating from a wide area^53^.

Within this facies succession—excluding sediment deposited near the calving line (see^63^)—productivity indicators, e.g., total organic carbon (TOC), ratios of carbon and nitrogen (C/N), absolute diatom abundances (ADA) and biogenic silica, are assumed to be low, and widely employed to differentiate between ice shelf, tidewater and open-marine depositional settings (Fig. 4). Diatoms cannot survive beneath ice shelves, except under special circumstances (see refs. ^19,70^). In contrast, a tidewater calving front would allow both GL proximal and diatom sedimentation to occur contemporaneously, giving rise to higher ADA/biogenic content, if the related siliciclastic sedimentation rates are low. Consistently low TOC content and ADA throughout sediment cores recovered from the seafloor below the former Larsen B Ice Shelf were used to demonstrate its persistence during the Holocene^17^ (Fig. 4), while increases in ADA has been associated with past ice-shelf retreats^71^. An exception to this general pattern occurs where diatoms and foraminfera are advected beneath the ice shelf from the open ocean^72–74^. High ADA in seafloor surface sediments beneath the Amery Ice Shelf^42^ (Fig. 4) are consistent with its modelled cavity oceanographic circulation^75^, providing a proxy for inflowing and outflowing water masses^42^.

In contrast to siliceous microfossils, variations in foraminiferal assemblages have been directly associated with ice shelf cover. Additionally, because ice shelves restrict air–sea gas exchange, stable carbon isotope (δ^13^C) values in benthic and planktic foraminifera tend to be lower in shells calcifying in sub-ice shelf waters^72^ relative to open-marine settings. Recent work in the Amundsen Sea suggests that a switch from a benthic foraminifera assemblage dominated by *Nonionella bradii* and *Globocassidulina* species (*Globocassidulina subglobosa*, *Globocassidulina biora*) to one composed of various *Angulogerina* species, and specifically *Angulogerina angulosa*, reflects a change from sub-ice-shelf to open-marine deposition (Fig. 4)^72^. Assemblages dominated by *G. subglobosa, G. biora, Trifarina earlandi, Astrononion echolsi* and *Nonionella* sp. have also been associated with sub-ice-shelf environments in the Ross Sea and Marguerite Bay^59,76,77^ while *Trifarina angulosa* (as *Angulogerina earlandi*) is associated with an ice-shelf edge environment in the Larsen Ice Shelf area^78^. Sub-ice-shelf sediments in the Whales Deep Basin, eastern Ross Sea (Fig. 1) have revealed two different morphotypes of *G. biora* (pustulose and smooth) as well as spinose and costate morphotype of *T. earlandi*^77^. The pustulose morphotype of *G. biora* appears to be most prominent in GL-proximal sediments and particularly those associated with GZW foresets (Fig. 4). One suggestion is that the smooth morphotype is more characteristic of distal sub-ice-shelf conditions while the pustolose is indicative of GL-proximal sedimentation, perhaps associated with an ice cliff^77^. The two morphotypes of *T. earlandi* strongly dominate the ice-shelf breakup facies, which might record benthic foraminiferal colonisation after significant habitat disturbance. Within the sub-ice shelf foraminifera assemblage in Pine Island Bay, there was also a high proportion of the non-encrusted shell morphotype of *Neogloboquadrina pachyderma* sinistral (adolescent stage), which the authors related to advection beneath the ice shelf and subsequent mortality before reaching the terminal life stage^72^. Minzoni et al.^74^ also propose advection to explain increases in *N. pachyderma* sin. in glacier proximal sediments deposited beneath an expanded Cosgrove Ice Shelf. Arenaceous benthic foraminifera are also common in ice shelf covered areas although their taxonomy and ecology is poorly known compared to other groups^79^, and their relative abundance in some settings might relate to preservation potential^77,80^. Nevertheless, *Miliammina arenacea* and *Portatrochammina* sp. have been documented in sub-ice-shelf sediments in Marguerite Bay^59^, Whales Deep Basin^77^ and Lallemand Fjord^81^, while *Portatrochammina pseudotriceramata* and *Cyclammina pusilla* have specifically been linked to grounding line-proximal sub-ice-shelf sediments^59^ (Fig. 4).

### Landforms

**Fig. 6:**
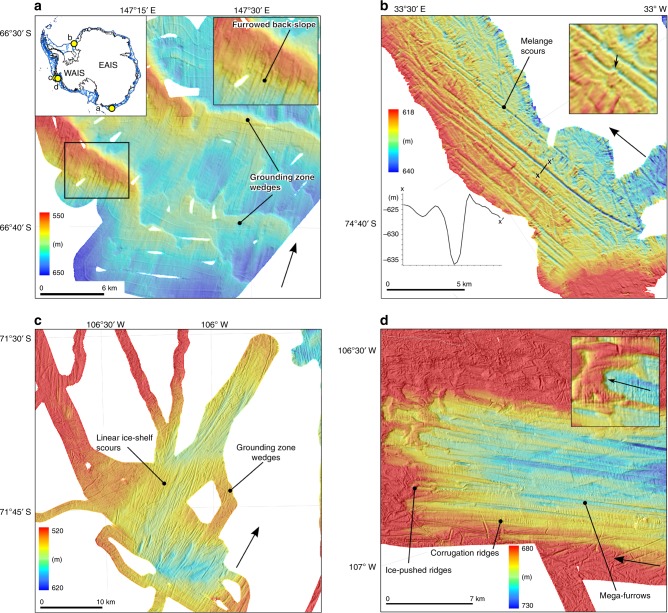
Landforms associated with ice shelf presence and absence. **a** Large grounding zone wedges crossing the Mertz Trough (30 m grid cell size), indicative of grounding line positions constrained by the presence of an ice shelf^148^. The backslopes of the wedges (inset, top-right) are marked by sub-parallel furrows that may have formed during ice-sheet lift-off. Map inset (top-left) shows location of panels **a**–**d**. −700 m (dark blue) and −500 m (light blue) bathymetric contours are shown to illustrate general continental shelf morphology. Colours and corresponding scales depict depth below sea level in metres in panels **a**–**d**. **b** Melange scours (Filchner Trough, 25 m grid cell size^91^) indicative of ice-shelf presence, particularly their sub-parallel geometry. Inset highlights an along-track ‘kink’ in the scours which shows they are not subglacial bedforms. **c** Linear ice-shelf keel scours on the tops of grounding zone wedges in outer Pine Island Trough (30 m grid cell size) recording the transition from grounded ice-sheet to floating ice-shelf^93^. **d** Range of landforms associated with retreating ice shelves (Pine Island Trough, 20 m grid cell size^93^), including corrugation ridges, mega-furrows and ice-keel plough ridges. Inset shows one of the ice-pushed ridges in detail. Black arrow (inset) is direction of ice keel motion to generate ice-keel plough ridge.

Supporting the sedimentary imprint of ice shelves, landforms, through an understanding of their geometry and geomorphic formation, provide additional records of ice shelf presence and absence. However, the landforms diagnostic of ice shelves are poorly characterised, reflecting both an under-appreciation of their potential but also technological limitations associated with sonar imaging systems that traditionally have been unable to resolve key features beneath the resolution of shipborne instruments (>20–30 m in Antarctic shelf depths). Landforms associated with ice shelf presence include grounding zone wedges (GZWs), various scours, corrugation ridges and moraines (Figs. 5 and 6; Table 1).

**Table 1:** Summary of geomorphological features associated with ice shelf presence and absence^30,83,84,91,93,112,149–151^.

	Landform type	Description	Dimensions	Formation/Interpretation	Examples
	**Landforms associated with ice-shelf presence**				
	Grounding zone wedges (GZW) [22]	Sedimentary wedges formed of soft diamicton (till), with an asymmetric cross-sectional geometry; internal ‘prograding’ reflectors and truncated topsets	Up to 15 km long, several metres to 100 m thick	Quasi-stable period when the ice margin remains near-stationary allowing the accumulation and forward progradation of a sedimentary wedge. Vertical restriction and elongated ‘wedge’ cross-profile indicate limited accommodation space due to the presence of a seaward floating ice tongue	Pine Island Trough^93,149^
	Melange scours [23]	Straight and sub-parallel, down-wardly incised linear landforms sometimes with dog-legged kinks along their tracks (Fig. 6a)	1–10 km long, ~100 m wide, and 2–10 m deep	Keel-mark incisions from a free-floating mass of icebergs held within an ice melange. The forward motion of the melange is aided by a floating ice shelf forming the seaward extension of an ice stream upstream	Filchner Ice Shelf, Weddell Sea^91^
	Sub-ice shelf keel scours [24]	Arrays of splayed and cross-cutting lineations with high linearity but low parallel conformity, typically on the surface of a GZW. In contemporary settings, scour terminates abruptly at a positive asymmetric mound	~100–400 m spacing/width, 1–10 km long, 1–10 m high	Scours formed as a marine ice stream comes afloat in the grounding zone. Terminal mounds created by sediment piling ahead of the forward-moving ice- shelf keel	Outer Pine Island Trough, West Antarctica^149^; Modern Pine Island Glacier ice shelf cavity^40^
	Corrugation ridges [25]	Small-scale transverse ridges, within or overprinting linear scours	0.5–2 m high, 70–150 m crest spacing, possible cyclicity in amplitudes	Expression of tidal lifting and settling of the ice shelf. At Pine Island Glacier, corrugations are interpreted as formed under the intact ice shelf based on: (1) observations that the sub-ice shelf ridge is a regular site of modern unpinning and regrounding by deep-drafted ice keels (2) Convincing sediment core evidence for full ice sheet grounding on the top of Jenkins ridge as recently as 1940s, and subsequent cavity opening, implying the surface morphology of the Jenkins Ridge is a fresh imprint of ungrounding. (3) A lack of direct geological evidence that the Pine Island ice shelf was absent during previous warm times of the Holocene, required to explain the corrugations as iceberg-formed features (although see ref. ^143^)	Modern Pine Island Glacier ice shelf cavity^30^
	Ice-shelf moraines	Gently inclined shore or ice-shelf edge parallel ice- cored linear debris accumulations formed around ice shelf edges	—	Originate from thrusted slabs of glacimarine sediment, folded debris- rich basal ice, and/or the accretion of sea-water and basal marine sediments. Sometimes contain marine organisms that can be dated	George VI Ice Shelf^83^; McMurdo (Minna Bluff), Sorsdal Ice Shelf^84^
	**Landforms of retreating ice shelves**				
	Iceberg ploughmarks [26]	Cross-cutting, curvilinear to sinuous scour marks. V- shaped in profile often with flanking berms. Sometimes terminate in rimmed pits. Single or multi-keeled expressions	10–>200 m wide, 100 s of m to >30 km in length, 1–>30 m deep	Ploughed grooves formed by scour from iceberg keels. Occur in a number of settings but expected to be abundant on seafloor landscapes shaped by ice shelf retreat. Cut-off in population depths potentially indicative of MICI processes	Pine Island Trough^112^. Norwegian margin^150^
	Ice-plough ridges [27]	Crescentic mounds at the termini of linear ice-keel furrows (Fig. 6b, inset)	<1 km long, 50–~200 m wide, up to ~20 m high	Formed by sediment pushing due to the multiple grounding of mega- icebergs during an ice-shelf breakup. Ridges or pits form prior to rotation and ungrounding of the berg keel	Mid-Pine Island Trough^93^; Northern Barents Sea^151^
	Mega-berg furrows [28]	Down-wardly incised deep, linear to slightly curved scours, sub-parallel	2–10 km long, 5–>20 m deep, 150–> 500 m spacing	Produced by deep keels of large and thick icebergs entrained in ice melange formed by rapid ice-shelf breakup	Mid-Pine Island Trough^93^; Northern Barents Sea^151^
	Corrugation ridges [25]	Small-scale, transverse ridges, forming tracks within or which overprint linear scours	0.2–2 m high, 35–200 m crest spacing, reduce in spacing down-flow. Amplitudes vary systematically along flow	Formed at the trailing edge of mega-bergs entrained in a coherent proglacial ice melange, retaining an expression of tidal lifting and settling of berg keels. Cyclicity in amplitude consistent with modulation by tides in open water, with one corrugation ridge forming per day by the gradual rising and settling of the iceberg keel on seabed sediments. The spacing of the ridges which reduces down-flow suggests the icebergs slowed as they drifted from the retreating ice face and grounded on the seaward-shallowing seabed	Mid-Pine Island Trough^93^; Northern Barents Sea^151^

Number in parenthesis (left column) equates to landform elements in Fig. 5.

Ice shelf moraines—formed where debris is deposited or redistributed around the ice-shelf margins—provide constraints on ice shelf presence and thinning history^82–85^ yet are only preserved in a handful of ice free terrestrial settings. Push moraines with similar dimensions to those observed beneath Mackay Glacier (~2 m) have also been imaged on deglaciated parts of the western Ross Sea shelf (e.g., JOIDES Trough). Their formation has been associated with push and squeeze in basal crevasses as well as delivery and deposition from sub- or englacial material^26,86^ although no clear association between these features and the presence of an ice canopy was found^86^. As such it remains unclear, if such moraines are diagnostic of ice shelf presence or simply indicate a specific GL configuration and/or retreat pattern.

GZWs form when the GL remains near-stationary allowing the accumulation and forward progradation of sediment to build a wedge geometry (Figs. 5 and 6a)^86–88^. Their vertical restriction and asymmetry implies limited accommodation for sediments above the grounding zone, due to the low seaward slope of an extending, floating ice tongue^88^. In the absence of a restricting ice shelf, flux of subglacial sediments will more likely construct morainal banks or proglacial grounding-line fans, both of which are distinct in their morphology and internal facies from GZWs^28^.

Geophysical data indicate that the under-sides of modern ice shelves display an irregular topography characterised by crevassing, longitudinal channels, terracing and discrete keels of varying depth^89,90^. It is likely that ice shelf keels regularly interacted with the tops of GZWs, where GL migrations occurred. Such ‘keel’ expressions are perhaps the most prominent landform signature discerning past ice shelf extents and create both indirect and direct evidence for ice shelves in the form of melange scours and sub-ice shelf keel scours. Melange scours (Fig. 6a) are highly linear keel-forms created as part of a free-floating mass of icebergs held within an ice melange (Fig. 5)^91^. On the seafloor in the Filchner Trough, a glacially-incised trough extending below and seaward of the Filchner Ice Shelf^91^ (Fig. 1), the forward motion of the melange was interpreted to have been aided by an ice shelf that formed the seaward extension of a large paleo-ice stream. Ice shelf advance pushed the floating ice melange that in turn scoured the sea floor beyond the calving line (Fig. 6b).

Sub-ice shelf keel scours (Fig. 5) are similar to, but genetically distinct from, melange scours and consist of splayed and occasionally cross-cutting lineations with high linearity but low parallel conformity. They have been imaged on the surfaces of relict GZWs^30^ and interpreted as erosional keel-imprints formed by the lift off of marine terminating glaciers^92^ (Fig. 6c). They record the transition from a grounded to a sub-ice shelf environment and form an intermediary landform between curvilinear iceberg ploughmarks, characterised by restricted lengths without a preferred direction, and long elongate subglacial bedforms which are unidirectional and highly parallel (Table 1). Indeed, linear furrows on the backslopes of many GZW that have previously been classified as subglacial may be re-interpreted as sub-ice shelf keel scours formed during decoupling of the ice bed with the grounding zone feature (e.g. Fig. 6a). We hypothesise that such sub-ice shelf keel-scours form in newly opened ice shelf cavities where ice reaches flotation.

The only high-resolution (2-m grid cell size) bathymetry from beneath an extant ice shelf, where such a lift-off is known to have occurred, were obtained with Autosub from the top of Jenkins Ridge beneath PIG ice shelf. Graham et al.^30^ interpreted seafloor scours to result from keel ploughing beneath the ice shelf as it passed over the ridge. The scours also contain populations of ‘corrugation ridges’; the expression of tidal lifting and settling of the ice shelf during scour formation. Identical corrugation ridges were also mapped in the outer part of Pine Island Trough. By contrast, these were interpreted to have formed as the expression of tidal motion in free-floating icebergs entrained in an iceberg melange (a collapse scenario)^93^. Multiple lines of evidence indicate that, at least beneath PIG, corrugations record sub-ice shelf keel interaction with the seabed and not past iceberg grounding in open water (Table 1)^30^. Indeed, it is likely that all the cavity geomorphic features on Jenkins Ridge relate to sub-ice shelf processes during the last century—perhaps even the last decade^21^. Thus in this setting, ice-shelf keel scours and associated corrugations provide direct evidence for ice shelf presence (Fig. 5a) although it remains to be seen whether they can be used as landform indicators for past ice-shelf presence more widely.

## Signature of ice shelf collapse

### Sediment facies and proxies

The collapse of ice shelves in the Antarctic Peninsula during the 1990s and early 2000s not only allowed a detailed examination of sediments deposited beneath ice shelves but also the marine geological signature of ice-shelf collapse. A key finding was that sedimentation rates increased by two to four times during disintegration (Fig. 4)^94^ due to deposition of sediment from the ice shelf surface and increased rainout of englacial and basal debris from the ice-shelf base (Figs. 4 and 5c, d). The geological diversity of IRD also increased significantly during collapse^95^, primarily due to icebergs floating freely and being sourced from a larger catchment than pre-collapse. A distinctive feature of the Larsen B Ice Shelf collapse was that aeolian sediment was concentrated in melt ponds on the ice shelf surface prior to its breakup (Fig. 5c). These ponds are held responsible for weakening the ice shelf via hydrofracturing^7^, leading to catastrophic drainage and release of the aeolian sediment to the seabed (Fig. 5c). Repeated recharge and drainage of the ponds over several years prior to collapse was archived in the seafloor sediments as multiple layers of aeolian sand. Above the aeolian sands, gravel-rich sediments are attributed to iceberg-dumped material entrained from medial moraines (Fig. 5d). Gilbert and Domack^94^ cautioned that both the sand- and gravel-rich deposits are likely to occur in patches and lenses rather than as an ubiquitous drape and that local source geology was important in the observed signature, perhaps creating a bias in the sand-sized fraction. Nunataks local to the Larsen ice shelves comprise easily frost-weathered Cretaceous sandstones. Accordingly, such ice shelves are more likely to preserve an ‘aeolian’ signal of disintegration relative to others.

Collapse of Larsen B also resulted in significant increases in ADAs in post-collapse sediments (Fig. 4)^17,96,97^. Other parameters, such as contents of biogenic silica did not increase substantially following its retreat^98^ although this might simply reflect the regional signature of opal, which tends to be low even in the open ocean. Although overall contents did not increase, a clear partition in the source of biogenic silica was documented^98^ with contributions from sponge spicules being higher within sub-ice-shelf sediments compared to post-collapse sediments. This reflects the capability of certain sponges (Hexactinellidae and Demospongiae) to survive under the outer limits of an ice shelf^34^. Biogenic detritus reaching the seabed after collapse was dominated by diatoms, and concomittant increases of chlorophyll pigments^65^. Sub-ice-shelf sediments, on the other hand, contain older and more refractory organic matter thought to be related to advection^65^. In this context, geochemical analyses that can distinguish different organic carbon compounds may provide a more powerful discriminator in reconstructing ice shelf cover and absence in the geological record. There is also untapped potential in using sponge spicules to help distinguish phases of ice shelf presence/absence—and particularly collapse—as sponges rapidly colonise the seabed following ice shelf disintegration^99^. In addition, work from the eastern Ross Sea also noted an increased abundance of freshwater diatoms (Chrysophytes) in sediments associated with a post-LGM ice-shelf breakup^73^. This was interrupted as a biotic signature of freshwater melt ponds which existed on the ice-shelf surface prior to the ice-shelf disintegration. *Fursenkoina fusiformis*, which is an opportunistic (calcareous) benthic foraminfera often associated with organic-rich sediment and oxygen-depleted waters, has been documented in ice shelf collapse facies recovered from Marguerite Bay^59^. Majewski et al.^77^ also document a strong association between the two morphotypes of *T. earlandi* and the ice shelf breakup facies described from Whales Deep Basin (Fig. 4).

The insensitivity of certain biological proxies, e.g., biogenic silica and TOC, to ice shelf collapse reflects both the influence of strong advection from the open ocean under an ice shelf—which tends to blur differences between ice shelf presence and absence—as well as other environmental factors supressing productivity, such as perennial sea-ice cover following collapse. Several studies show that concentrations of beryllium isotopes (^10^Be) vary between subglacial, sub-ice shelf and open marine sediments (Fig. 4), leading to its use as a proxy for ice shelf cover^100–102^. Cosmogenic ^10^Be is produced at an essentially constant rate in the upper atmosphere by cosmic radiation, and is transferred to the surface ocean by precipitation, where it is quickly adsorbed to terrigenous clay and other particles, that rain out to the seafloor. One complicating factor is that radiogenic beryllium is adsorbed to diatom frustules^103^ and so its signature in sub-ice-shelf sediment can also relate to lateral transport from areas of open water directly in front of an ice shelf^104^. More recent work^104^ suggests that the HCl-extractable ^10^Be/^9^Be ratio might provide a more robust discrimination of sediment deposited in open marine and sub-ice shelf settings, as this can help distinguish between the various pathways of Be.

The potential of offshore sedimentary successions (continental slope, rise and deep-sea) for reconstructing ice shelf history is comparatively understudied, due to the complexity in interpreting ice-distal records. Accumulation rates^105,106^ and/or provenance^107^ of coarse-grained IRD can be a useful proxy for detecting ice mass loss in Late Cenozoic far-field records, with microtextures of these grains even allowing distinction between subglacial, englacial or supraglacial transport^108^. However, periods of ice shelf loss cannot be easily disentangled from normal calving and/or retreat of ice streams unless allied with accurate near-field sea-level data; ice shelf collapse is likely to have minimal impact on sea-level whilst ice sheet collapse will, although this is also dependent upon the impact of ice shelf removal on terrestrial ice. Ice shelf collapse might also leave an imprint on the global δ^18^O signal in benthic foraminifera, and has been used to explain rapid δ^18^O decreases following the LGM and the penultimate glaciation^109^. This argument has been used to support the idea of a pan-Arctic Ocean ice shelf during Marine Isotope Stage (MIS) 6 ka^110^. Here, the amplitude of the glacial–interglacial δ^18^O shift in the deep-sea benthic foraminifera record predicts more ice volume than available sea-level records indicate, a discrepancy that can be explained if ^16^O is stored in a large ice shelf that, once melted, has only a minor effect on sea level^111^.

### Landforms

A defining characteristic of landforms associated with retreating ice shelves is the interaction of ice keels with the seafloor (Fig. 5a, b). Some suites of iceberg ploughmarks almost certainly relate to past ice-shelf retreat phases^112^. However, these landforms are still being created in Antarctica today, making it difficult to discern “fossil” from modern ploughmark populations, particularly in shallower shelf regions, whose water depths overlap with keel depths of icebergs calved from modern ice shelves (<650 m^113^).

A more useful set of diagnostic landforms occurring in the shallow shelf areas have been termed ‘ice plough ridges’ (Fig. 6d). Ice plough ridges are formed by seafloor sediment pushing due to the multiple grounding of icebergs during an ice-shelf breakup^114^. These bedforms are generally restricted to sea beds that shoal gradually seaward from the ice front. Their relationship to ice-shelf retreat is linked to the idea that a distinct population of plough ridges form near synchronously during a single event, but the ridges are usually distal to the inferred calving front and only occur under circumstances in which the retreating ice shelf front is thick enough to calve icebergs with keels of suitable draft to ground at the sea floor. Therefore, ice plough ridges may not always be created, even during large ice-shelf collapse events.

Similar glacial features to linear melange scours described previously have been associated with episodes of past ice-shelf breakup on the middle continental shelf of Pine Island Trough^93^. Unlike other streamlined subglacial bedforms, these ‘mega-berg furrows’ have a pronounced relief (>20 m), and are characterised by clear downward incisions at irregular spacings, indicating they did not form subglacially. Jakobsson et al.^93^ interpreted these scours as having formed by an armada of grounded icebergs discharged during ice shelf breakup and retreat. Where found, these landforms may represent the proglacial signature of ice shelf collapse and may indicate ice-shelf extent and width, ice-shelf thickness during retreat, keel spacing and configuration, and even relative timing of the evolution of the collapse event in cases where they crosscut pre-existing morphological features. Corrugation ridges have also been used as diagnostic landforms of ice-shelf retreat and absence (Fig. 6d). In Pine Island Trough, corrugations form tracks of 1–2 m high ridges at the seabed, overprinting mega-berg furrows, with indicators for tidal modulation in their amplitudes and patterns suggesting that icebergs with deep drafts had been entrained in a large ice melange seaward of the ice shelf front (Fig. 6d). The assemblage of landforms in Pine Island Trough, including corrugations^93^, thus record the complete collapse of an ice shelf back to the grounding line. Therefore, corrugations can be useful diagnostic indicators of widespread ice-shelf collapse. If dated, they can reveal the pace at which icebergs are shed from the ice shelf, as well as the speed at which the ice melange carried the bergs forward (c. 50–150 m per day).

Part of the model for the formation of corrugation ridges, and of the signature of collapsing ice shelves, involves the scenario that icebergs can calve to full-depth near the GL (Fig. 5)^93^. Subsequently, this mechanism has been discussed and expanded upon in theoretical studies: where ice-shelf retreat leads to the exposure of thicker inland ice without a buttressing floating tongue, a positive feedback of structural failure of the ice cliff has been proposed (termed ‘marine ice cliff instability’; MICI). MICI has been incorporated into ice sheet models and constitutes a key physical process by which ice sheets rapidly disintegrate^15^. Wise et al.^112^ showed that this mechanism may have occurred during post-LGM retreat of collapsing ice shelves in the Amundsen Sea, with its signature preserved in the shapes of ploughmarks carved into the sea floor. Structural physics of ice shelves suggests that the landform signatures of MICI should only be present at discrete water depths^112^. However, the mechanism remains poorly studied and presents a challenge for geoscientists to discover, if MICI leaves additional landform signals at the sea floor.

## Establishing past ice shelf and cavity geometry

Establishing the thickness and extent of former ice shelves is critical for ice sheet mass balance calculations because the flux of ice across the GL increases strongly as a function of its thickness. Ice shelf thickness at the GL is quantifiable (for a single point in time) using past sea-level estimates that are corrected for isostatic rebound^115^ coupled with depths of GZW crests at the sea floor. Relative elevations of GZW (e.g., Pine Island Trough) also indirectly indicate past ice-sheet surface profiles, which in turn can be used to infer paleo-ice-shelf surface profiles. At the Pine Island-Thwaites paleo-ice stream bed, the GL was elevated by only ~80 m during a retreat of more than 100 km, implying a very low-gradient ice stream. GZWs do not give a clear indication of the calving-line location of an ice shelf but this can be inferred by analysis of the sedimentary record if the density of sediment cores is sufficient to map the spatial distribution of ice shelf/calving line facies. However, with the exception of a few targeted areas, such datasets are rare on the Antarctic shelf. Establishing the extent of ice shelves using the sedimentary record also requires good-dating control to ensure that sediments were deposited contemporaneously, and this often represents the biggest challenge.

Constraints on past cavity geometry come from both seabed sediments and geomorphology, and by necessity require detailed bathymetric data which are currently unavailable for large areas of the Antarctic seabed, less so beneath extant ice shelves. Cavity geometry strongly influences the transfer of heat to the ice sheet^10^, and hence knowledge of its shape is important for understanding the processes that determine melt and thus ice-shelf sensitivity to external forcing. As an ice shelf base limits the height of a GZW, cavity size can be estimated if the bathymetry seaward of the GZW is known.

Indirect information about the evolution of the cavity can be inferred from the distribution of sedimentary facies seaward of a GZW. On a landward sloping bed, GL retreat results in a gradual enlargement of a sub-ice-shelf cavity. Although governed by several interacting variables, modern observations indicate that the majority of debris melts out from the ice shelf base within 1.5–10 km of the GL^23,24^, meaning that the transition from coarse-grained GL-proximal sediments to more distal finer-grained muds can be used as a crude yet meaningful estimate for former positions of the GL^16^, and by inference cavity geometry. Cross-bedded and/or laminated muds directly above the coarse-grained facies also indicates deposition in a shallow cavity. Because tidal pumping creates traction currents that influence particle sorting, changes in current strength(inferred from sortable silt) may be indirectly associated with the change in cavity shape and associated weakening of tidal-pumping processes^20,63^.

Finally, it is worth reiterating that there exists no single diagnostic tool for identifying ice shelf presence, rather a succession of sedimentary facies and proxies that are linked to the proximity of the GL and calving line and the interplay between inflowing and outflowing currents. Likewise, the landform record is often patchy and in some instances poly-genetic, whereby certain landforms (e.g., corrugation ridges) can form in more than one setting. This highlights the need to apply a multi-proxy approach and integrate the geomorphological context in order to reconstruct ice shelf presence, collapse or re-growth^25,116^. In instances where the sedimentary and/or landform record is sparse or inconclusive, modelling might offer additional support for former ice shelf cover^117^.

## Constraining rates of change

Establishing the duration of past ice shelf cover, timing and rates of ice-shelf retreat requires accurate chronologies. Unfortunately, the variable but predominantly terrigenous nature of sediments deposited in a sub-ice-shelf setting combined with low concentrations of biogenic material (organic carbon and calcareous foraminifera) represent significant challenges for conventional dating methods. On short times scales (<200 years), measurements of the concentrations of short-lived radioisotopes, such as ^210^Pb, have helped constrain GL retreat, as well as recent sedimentation rates^16,17,96,118^. While fallout of atmospheric lead is typically preserved around Antarctica^16,119^, concentrations of other radioisotopes, such as ^137^Cs and ^241^Am, which are required to validate ^210^Pb decay profiles, are highly variable and often below analytical detection limits. ^137^Cs—injected into the atmosphere during nuclear bomb testing in the 1950s and 1960s—has a half-life of only 30 years, and in many Antarctic settings has already decayed below detectable levels. Furthermore, ^137^Cs is especially problematic in the Southern Hemisphere because fallout levels were approximately four times lower than in the Northern Hemisphere^120^. Smith et al.^16^ demonstrated the potential of using Pu-isotopes to validate ^210^Pb age models in sub-ice-shelf sediments. Plutonium isotopes, in contrast to other bomb-test products, have a much longer half-life and are expected to adsorb more readily than Cs to both coarse- and fine-grained detrital particles, with little chance of chemical re-mobilisation once the Pu is buried^121^.

On longer times scales (up to ~35,000 years) AMS ^14^C dating of calcareous microfossils provides the most reliable chronologies from marine sediments^59,72,116^ and can now be performed on very small sample sizes^122^. Unfortunately, the scarcity of such microfossils (even in low concentrations) in many sub-ice-shelf sediments has forced researchers to rely on dating other fractions^123,124^. Bulk organic matter or its acid-insoluble fraction (AIOM) can yield reliable and reproducible chronologies (see ref. ^124^), but it is often viewed as a last resort owing to the potential for large error caused by “contamination” with reworked fossil carbon^125^. Recent advances in Ramped Pyrolysis (RP) ^14^C dating minimises the effect of contamination by combusting sediments at gradually increasing temperatures to thermally break down the bulk organic matter^64,126,127^. The gradual temperature increase allows for the separation of the more thermochemically reactive younger constituents, which are preferably combusted under lower temperatures, from the reworked more stable older constituents, so that separate components can be dated independently^127^. Similarly, chemical compound-specific radiocarbon dating enables specific organic compounds (C14, C16 and C18 fatty acids) to be isolated, and thereby avoiding fossil carbon. Fatty acids in the surface waters of the Southern Ocean are primarily produced by diatoms and because their decomposition is relatively fast in both the sediment and water column^128,129^ they rarely stem from relict organic matter. Therefore, the majority of fatty acids extracted from sediments are formed just before deposition, rather than being derived from relict organic matter^130^.

Both RP and chemical compound-specific dating rely on a minimum concentration of organic carbon which is not always present in sub-ice-shelf sediments. In instances where sub-ice-shelf sediments are devoid of biogenic material or contamination with fossil organic carbon is high, relative paleomagnetic intensity dating offers an alternative for establishing reliable age models for Holocene sediments^71,131^. However, this method is susceptible to changes in magnetic mineralogy and magnetic grain size, and thus to sediment provenance and physical grain size, both of which can vary considerably in sub-ice shelf sediments. Nevertheless, Brachfeld et al.^71^ used this technique successfully to constrain the timing of Holocene retreat of the Larsen A Ice Shelf, demonstrating its potential for dating mainly fine-grained terrigenous sediments deposited beneath an ice shelf. Finally, in regions where aeolian sand and silt accumulates at the calving line and on the ice shelf surface until being released during collapse (Fig. 5a, d), multiple-grain optically stimulated luminescence (OSL) dating has shown some promise^132^, although this technique is unlikely to provide the resolution needed to constrain centennial to millennial-scale environmental changes.

## Drivers of ice shelf retreat

Placing ice shelf histories in the context of climate drivers is needed to constrain the interplay between internal ice sheet dynamics and external forcing. Ice cores provide a valuable archive of environmental change from which it is possible to infer past atmospheric temperatures, precipitation and changes in atmospheric circulation, and previous ice-shelf retreats have been linked to periods of atmospheric warming. Similarly, clues to the behaviour of ocean currents and specifically the role of upwelling of warm CDW in driving ice shelf melting can be derived from a number of proxies, each with its own uncertainty and challenges. Benthic foraminiferal assemblages have been used to infer incursions of CDW, although results have often been contradictory^133,134^ and likely reflect an incomplete understanding of ecology and habitat preferences of key species. Traditionally *Bulimina aculeata* has been considered to be an index species for CDW^81,135,136^ and has been well documented in surface sediments in the Amundsen Sea that are currently bathed in this water mass^137^. Despite this association, *B. aculeata* is almost entirely absent in surface and Holocene down-core sediments recovered from Pine Island Bay, where CDW is present today and assumed to have been present in the past^72^.

Other attempts to reconstruct CDW intrusions have focussed on its geochemical fingerprint^138^, as well as stable carbon isotope composition of benthic foraminifera^72,134^ or indirectly using a multi-proxy approach^139,140^. Efforts to reconstruct ocean temperatures directly offer huge potential, yet are still in their infancy around Antarctica^72,139,141^, lacking both regional calibrations and well-dated records on which to apply temperature proxies. Without accurate paleocean temperatures the significance of current sub-ice-shelf melt rates remain difficult to validate, although information on past ice shelf melt rates can be derived from δ^18^O studies. Pike et al^142^ demonstrated that it is possible to infer glacier discharge (melt) using δ^18^O measurements in diatom silica, although problems remain with disentangling ice shelf melt from other sources of glacial/freshwater discharge or chemical processes. δ^18^O data from planktic foraminifera have been used to infer water column freshening due to the long-term thinning of the Larsen B Ice Shelf^17^. Needless to say the usefulness of this proxy—together with benthic foraminiferal assemblages and trace metal and stable isotope data from calcareous shells—is dependent on preservation of foraminifera, which is often poor in sub-ice-shelf environments.

## Outlook and future research

The past decade has witnessed significant progress in our understanding of sub-ice-shelf environments, resulting in a comprehensive ‘toolbox’ with which to identify past ice shelf cover as well as passive retreat and/or breakup events. This not only reflects technological advances in remote access but also detailed analyses of sediments (Fig. 4) and landforms (Figs. 5 and 6) exposed following contemporary ice shelf collapse(s)^58,65,94–97^. Together with recent advances in dating^64^ and multi-proxy approaches^72^ this work holds the potential to usher in a new era of deciphering former ice shelf cover together with the rates and drivers of change. To enable this, there needs to be a concerted effort to link the often disparate strands of research, including constraints on ice shelf thickness and extent from the geomorphological record together with detailed sedimentology, proxy reconstructions and accurate dating. Furthermore, because Antarctic continental shelf sediments are often lacking biogenic material (diatoms, foraminifera), greater progress can be made by re-visiting areas where the sediments are known to be suitable for re-analysis using the latest proxies and dating methods and where the geomorphological footprint of former ice shelf cover is well-preserved (e.g., Pine Island Bay^72^, Marguerite Trough^59^, Whales Deep^116^ e.g., Box 1).

Despite this progress, sub-ice-shelf settings remain one of the least explored environments on Earth, and there is a clear need to access and sample the seafloor and ocean cavity beneath contemporary ice shelves. This will not only help answer debates about ice shelf and GL histories at a range of time scales^16,20,143^ but also reveal fundamental information about the processes occurring beneath ice shelves, particularly those close to the GL. In this context, much of our knowledge of sub-ice-shelf sedimentary processes, particularly those operating at or close to the GL, has been obtained from a limited number of studies in the Ross Sea, which is almost certainly not representative of all ice shelf systems. Future work, while ambitious in scope, should aim to combine transects of sub-ice-shelf sediment cores from the GL to calving line, with down-hole oceanography and dedicated ROV/AUV missions (e.g., through-ice vehicles such as IceFin and SCNII or ship-launched platforms such as Kongsberg’s Hugin AUV) that image the seafloor, ice base and processes operating near the GL. Only by investigating the ocean cavity and seafloor beneath ice shelves of different sizes, experiencing different rates of change, and forced by different oceanic and atmospheric drivers, will it be possible to capture the full-range of processes operating there. The areas requiring further focus should include, but not be limited to, the spatial distribution of sediment facies/facies geometries, sediment and meltwater fluxes, seafloor benthos, circulation patterns and advection of detrital and biogenic particles as well as processes which form enigmatic landforms (e.g., GZWs, corrugations ridges). With planned multi-national expeditions to the Eastern Ice Shelf of Thwaites Glacier as well as further work on the Amery and Ross ice shelves over the coming years, our knowledge of these processes is set to expand considerably.

Finally, an important aim of paleo reconstructions is to provide highly resolved records of past changes that can be used to constrain and/or initialise numerical models^144^. This includes information on key boundary conditions (e.g., bathymetry, cavity geometry) as well as accurate constraints on the rates and drivers of ice shelf and ice sheet changes. However, integration of data and models is often intended but not always realised, which calls for a concerted effort to provide relevant paleo data in a form that can be easily utilised by modellers^144^. By producing detailed information on known past ice sheet-ice shelf configurations—such as complete loss of buttressing due to ice shelf collapse or stabilisation of a grounding line due to sediment deposition (see Box 1)—the paleo record can serve as a critical test for assessing the abilities of models in predicting future changes as well as refining our understanding of the processes which limit or promote fast retreat of marine ice sheets.

Box 1 The influence of ice shelf collapse on grounding line retreat

Detailed sedimentological and bathymetric data together with accurate dating of foraminifera illustrates a major phase of GL retreat following the collapse of an ice-shelf in Whales Deep Basin (WDB), eastern Ross Sea^116^. The study shows retreat was underway from the shelf edge prior to 14.7 ± 0.4 cal. kyr B.P. A paleo-ice-shelf collapse occurred at 12.3 ± 0.2 cal. kyr B.P. The GL was maintained on the outer-continental shelf until at least 11.5 ± 0.3 cal. kyr B.P before experiencing a 200-km retreat. GL retreat lagged ice-shelf collapse by at least two centuries and by as much as 14 centuries. This delay was due to rapid aggradation of sediment (GZW 5-7) as ice-stream discharge accelerated following ice-shelf collapse which helped stabilise the ice-sheet. This study serves as an example of how targeted coring, geomorphological mapping and reliable dating can provide well-constrained palaeodata with which to test the predictive skill of ice-sheet models and explore key processes, e.g., the dynamic response of ice-streams to ice shelf collapse and de-buttressing. One element that is missing from this study is information related to external and/or internal forcing which could be explored via numerical modelling and/or by applying multi-proxy analyses of nearby sediment cores to reconstruct key environmental variables (e.g., ocean temperature).
